# Randomized Clinical Trial of Intrapartum Clindamycin Cream for Reduction of Group B Streptococcal Maternal and Neonatal Colonization

**DOI:** 10.1155/S1064744996000063

**Published:** 1996

**Authors:** R. S. Gibbs, F. McNabb

**Affiliations:** 1 Department of Obstetrics and Gynecology Perinatal Clinical Research Center University of Colorado School of Medicine Denver CO USA; 2 Department of Pediatrics Perinatal Clinical Research Center University of Colorado School of Medicine Denver CO USA; 3 Department of Obstetrics and Gynecology University of Colorado Health Sciences Center 4200 E. 9th Avenue B198 Denver CO 80262 USA

## Abstract

*Objective:* In a randomized trial, we sought to determine whether 2% clindamycin cream administered intravaginally during labor to group B streptococcal-colonized pregnant women without risk factors would decrease maternal and neonatal colonization.

*Methods:* The eligible women were randomized to receive either cream or no treatment. Two hours after treatment or admission, the patients were tested with rectal and vaginal cultures. The neonates of the study patients were also tested.

*Results:* Of women randomized to cream, 5 of 5 remained positive and 2 (33%) of their 6 neonates were positive. Of 4 randomized to no treatment, 3 (75%) remained positive and 1 (25%) of 4 neonates was positive.

*Conclusions:* Intravaginal 2% clindamycin cream was ineffective in reducing colonization with group B streptococci.


Infectious Diseases in Obstetrics and Gynecology 4:25–27 (1996)
© 1996 Wiley-Liss, Inc.

### Randomized Clinical Trial of Intrapartum Clindamycin Cream for Reduction of Group B Streptococcal Maternal and Neonatal Colonization

**R.S. Gibbs and F. McNabb**

*Departments of Obstetrics and Gynecology (R.S.G.) and Pediatrics (F.M.), Perinatal Clinical Research Center, University of Colorado School of Medicine, Denver, CO*

ABSTRACT

***Objective:*** **In a randomized trial, we sought to determine whether 2% clindamycin cream administered intravaginally during labor to group B streptococcal-colonized pregnant women without risk factors would decrease maternal and neonatal colonization.**

***Methods:*** **The eligible women were randomized to receive either cream or no treatment. Two hours after treatment or admission, the patients were tested with rectal and vaginal cultures. The neonates of the study patients were also tested.**

***Results:*** **Of women randomized to cream, 5 of 5 remained positive and 2 (33%) of their 6 neonates were positive. Of 4 randomized to no treatment, 3 (75%) remained positive and 1 (25%) of 4 neonates was positive.**

***Conclusions:*** **Intravaginal 2% clindamycin cream was ineffective in reducing colonization with group B streptococci.** © 1996 Wiley-Liss, Inc.

KEY WORDS

Neonatal sepsis, pregnancy, GBS

The recent recommendations for the prevention of early-onset neonatal sepsis caused by group B streptococci (GBS) have been limited to parenteral intrapartum antimicrobial prophylaxis for women with clinical risk factors.[1,2] However, approximately 30% of the cases of early-onset GBS sepsis occur in pregnancies without risk factors. Prompted by recent work with hexachlorophene douches in labor,[3] we conducted a randomized trial to evaluate 2% clindamycin cream as an intrapartum topical approach in colonized women who did not have clinical risk factors for neonatal sepsis. We chose this cream because only 1–3% of GBS strains are resistant to clindamycin, because it is safe in pregnancy[4] and because it is commercially available.

### MATERIALS AND METHODS

This protocol was approved by our institutional review board. Under an existing clinical program of universal screening and selective intrapartum prophylaxis,[5] patients with positive cultures at 26–28 weeks were identified by a research nurse after their admissions in labor. The exclusions were age younger than 18 years, allergy to clindamycin, recent use of other antibiotics, and presence of a clinical risk factor for GBS neonatal sepsis [anticipated preterm birth <37 weeks gestation, preterm premature rupture of the membranes (PROM), PROM >12 h, and a clinical diagnosis of chorioamnionitis].[5] The eligible patients were invited to participate in a randomized trial of clindamycin cream (2%) vs. no treatment. After an informed consent was obtained

Address correspondence to Dr. R.S. Gibbs, Department of Obstetrics and Gynecology, University of Colorado Health Sciences Center, 4200 E. 9th Avenue, B198, Denver, CO 80262.

Received November 14, 1995
Accepted March 26, 1996

from the patient, a physician or research nurse collected separate sterile swabs from the distal vagina and the rectum. These were processed for GBS using selective media and semiquantitative technique.[5]

With a computer-generated randomization, the patients received either 5 ml of clindamycin cream (2%) intravaginally through an applicator, with up to 2 repeat doses, every 6 h in the absence of risk factors, *or* no treatment in a ratio of 2:1. Two h after each application of the cream or, in the patients with no treatment, after the patient's assignment, we repeated the rectal and vaginal cultures. If a patient developed a risk factor in labor, then we planned to give parenteral antibiotics and eliminate this patient from further consideration.

We obtained swabs from 4 neonatal sites (throat, ear, umbilicus, and rectum) and processed these for GBS, as above. The results were reported by the site of culture.

Because of the lack of data on eradicating genital GBS with clindamycin cream, some might suggest that these experiments should be carried out only in nonpregnant women. However, clindamycin cream is safe in pregnancy. Indeed, it is the CDC treatment of choice for bacterial vaginosis in the first trimester.[4] We were able to carry out a cost-effective study by attaching it to an existing protocol.

For sample size determination, we expected that 1) 65% of the women colonized at 26–28 weeks would remain positive on admission,[2] 2) the cream would reduce both maternal and neonatal colonization by 85–95%, and 3) GBS-positive cultures would persist in 85–95% of untreated patients in labor. We projected a sample size of 93 patients (62 receiving clindamycin cream and 31 no treatment) using a power of 0.8 and $\alpha$ at 0.05.

### RESULTS

From December 1, 1993, to August 31, 1994, 15 patients were enrolled; all were at ≥37 weeks gestation. At this point, we were aware that the rate of positive cultures after cream application was different from expected. We then carried out an interim analysis. Of the 15 participants, 5 (33%) were culture negative for GBS at admission. Four of these received cream and 1 no treatment. None of the remaining 10 received parenteral antibiotics prior to delivery. Of the 6 culture-positive women randomized to the cream, 1 did not have vaginal or rectal cultures obtained. Five of 5 with vaginal cultures and 3 of 5 with rectal cultures remained positive. One of these 5 women received multiple applications (3 doses). Her initial vaginal culture showed 4+ growth and the culture after the third dose was still positive with 1+ growth. The others delivered within 6 h of their first dose. Two of their 6 neonates (33%) were positive at 1 or more sites. One infant was positive at all 4 sites (with 4+ growth) and another had a positive umbilical culture (broth only) but negative cultures at the other 3 sites. Of the 4 culture-positive women randomized to no treatment, 3 (75%) remained positive vaginally and rectally, and 1 of 4 neonates (25%) was positive. This infant was positive at all 4 sites (with 4+ growth). These rates were not significantly different. At this point, we calculated that the probability of finding GBS eradication rate with the cream of approximately 0.9, with an observed rate of 0 of 5, is essentially zero. Therefore, we terminated the study prematurely.

### DISCUSSION

We found that 2% clindamycin cream applied vaginally in labor was ineffective in eradicating GBS vaginal or rectal colonization 2 h after the application. The rate of neonatal colonization after the cream application was not different from the rate after no treatment. Even though the numbers are small, we found that the observed rates of GBS eradication with clindamycin cream were far below expectations and impractical for clinical use.

Considering the reasons for the ineffectiveness of clindamycin cream, we may not have allowed enough time. However, for many patients, if delivery does not occur in a few hours, risk factors may develop. It is unlikely that the GBS isolates were resistant to clindamycin, but the amniotic fluid or fetus may have been colonizated before the application of the cream.

### ACKNOWLEDGMENTS

This work was supported by a grant from the Upjohn Company.

### REFERENCES

1. American College of Obstetricians and Gynecologists: Group B streptococcal infections in pregnancy. ACOG

Technical Bulletin No. 170. Washington, DC: American College of Obstetricians and Gynecologists, 1992.

2. Committee on Infectious Diseases and Committee on Fetus and Newborn: Guidelines for prevention of group B streptococcal (GBS) infection by chemoprophylaxis. Pediatrics 90:775–778, 1992.
3. Burman LG, Christensen P, Christensen K, et al.: Prevention of excess neonatal morbidity associated with group B streptococci by vaginal chlorhexidine disinfection during labour. Lancet 340:65–69, 1992.
4. Centers for Disease Control and Prevention, 1993 Sexually transmitted diseases treatment guidelines. MMWR 42:70, 1993.
5. Gibbs RS, McDuffie RS, McNabb F, Fryer GE, Miyoshi T, Merenstein GB: Neonatal group B streptococcal sepsis during two years of a universal screening program. Obstet Gynecol 84:496–500, 1994.
